# Fluorescence-guided lymphadenectomy in robot-assisted radical prostatectomy: the role of interventional radiology

**DOI:** 10.3389/fradi.2025.1548211

**Published:** 2025-03-12

**Authors:** Michele Usai, Emma Solinas, Claudio Fabio, Massimo Madonia, Alessandro Tedde, Giacomo Sica, Stefania Tamburrini, Salvatore Masala, Mariano Scaglione

**Affiliations:** ^1^ Department of Radiology, University Hospital of Sassari, Sassari, Italy; ^2^ Unit of Vascular and Interventional Radiology, Department of Radiology, University Hospital of Sassari, Sassari, Italy; ^3^ Department of Urology, Urologic Clinic, University Hospital of Sassari, Sassari, Italy; ^4^ Department of Radiology, Monaldi Hospital, Naples, Italy; ^5^ Department of Radiology, Ospedale del Mare-ASL NA1 Centro, Naples, Italy; ^6^ Department of Radiology, James Cook University Hospital & Teesside University, Middlesbrough, United Kingdom

**Keywords:** interventional radiology, prostatic neoplasms, sentinel lymph node biopsy, prostatectomy, indocyanine green

## Abstract

**Background:**

Bilateral extended pelvic lymph node dissection (ePLNR) is used in high-risk prostate cancer for assessing metastatic involvement and lymph node staging. Nevertheless, in patients with localized or locally advanced prostate cancer, loco-regional lymph nodes are not always metastatic. Based on this assumption, the aim of this study is to evaluate the potential of ePLND performed under fluorescence guidance after administration of the Indocyanine green (ICG)—Lipiodol mixture via embolization of the prostate arteries in order to identify metastatic lymph nodes, that are then confirmed by histopathology analysis.

**Materials and methods:**

All participants underwent selective embolization of the prostatic arteries 24–48 h before the scheduled surgery. The embolization procedure involved the injection of 25 mg/ml ICG, distilled water, and Lipiodol adequately mixed. During ePLND, the “Firefly” mode integrated into the Da Vinci robotic system was used to assess fluorescence in loco-regional lymph nodes. The lymph nodes were harvested and sent for histopathological examination. Intraoperative fluorescence results, histopathological findings, and short-term postoperative complications were recorded and classified according to the Clavien-Dindo system. For statistical analysis, the Phi coefficient was used to assess the correlation between categorical variables.

**Results:**

Ten patients diagnosed with high-risk or unfavorable intermediate-risk PCa were included. All patients underwent radical robot assisted prostatectomy with ePLND within 48 h of prostate embolization using ICG-Lipiodol. Intraoperative fluorescence results, final histopathological findings and postoperative complications were recorded. The lymph nodes with positive fluorescence, after being analyzed separately, were confirmed to be as metastatic upon dedicated histopathological examination, while non-fluorescent lymph nodes were found to be negative for metastatic involvement. The phi coefficient was calculated to establish the degree of correlation between detection of green fluorescence by Firefly system and the positivity of lymph nodes for metastatic invasion at the histopathological analysis. The concordance assessed by phi correlation coefficient was 0.76, with a sensitivity of 100% (95% confidence interval).

**Conclusion:**

Although preliminary, the results of this study demonstrate the potential of fluorescence-guided ePLND after ICG-Lipiodol administration for improving the identification of metastatic lymph nodes during Robotic-assisted radical prostatectomy RARP. Further studies are required to validate our findings with a larger group of patients.

## 1 Introduction

Prostate cancer (PCa) is the most common malignancy among men, though not the leading cause of cancer-related mortality (1). It is the second most frequently diagnosed cancer in men, with an estimated 1.4 million global diagnoses in 2020 (2). According to a review of autopsy studies, the prevalence of PCa is reported at 5% for individuals under 30 years of age, with an odds ratio increasing by 1.7 per decade, reaching a prevalence of 59% in individuals over 79 years old (3).

The term clinically significant PCa (csPCa) is used to differentiate cases that may lead to morbidity and mortality in specific patients, characterized by a substantial impact on the “quality and quantity of life,” from clinically non significant, often asymptomatic, cases (3). Without this distinction, many indolent tumors risk overtreatment, potentially causing harmful consequences for patients.

Abdominal computed tomography (CT) and magnetic resonance imaging (MRI) are commonly employed to evaluate suspected metastatic lymph node involvement indirectly, based on nodal diameter and morphology. However, the size of non-metastatic lymph nodes varies significantly and can overlap with metastatic lymph nodes. Suspicious characteristics include a round shape with a short axis ≥8 mm, an oval shape with a short axis ≥10 mm, heterogeneous appearance, and irregular margins. Lowering these threshold values improves sensitivity but reduces specificity, leaving the ideal threshold values uncertain (4, 5).

Positron emission tomography (PET) with Ga/18F-PSMA (Prostate Specific Membrane Antigen PET/PSMA offers a viable alternative due to its specificity for prostatic tissue, although its expression in other non-prostatic malignancies or benign conditions can result in incidental false positives. A multicenter study of patients with intermediate and high-risk PCa undergoing radical prostatectomy (RP) with extended pelvic lymph node dissection (ePLND) reported a sensitivity and specificity of 0.40 and 0.95, respectively, for 68Ga-PET/PSMA (6). A meta-analysis of 13 studies comparing PET/PSMA and MRI demonstrated higher sensitivity for PET/PSMA with comparable specificity between the two techniques in preoperative staging of lymph node metastases in intermediate- and high-risk PCa patients (7, 8).

PET/PSMA has shown good sensitivity and specificity in the preoperative staging of pelvic lymph nodes, influencing therapeutic decisions and strategies. However, despite its superior sensitivity compared to CT or MRI, the spatial resolution of PET/PSMA remains a limitation for identifying micrometastases.

Bilateral ePLND remains the gold standard for assessing metastatic involvement and lymph node staging in prostate cancer. Its use is indicated in high-risk disease and, following dedicated score calculations, in unfavorable intermediate-risk disease. Nevertheless, in patients with localized or locally advanced prostate cancer, loco-regional lymph nodes are not always metastatic. Moreover, as part of radical prostatectomy, ePLND is not free from complications and may represent overtreatment for many patients.

Indocyanine green (ICG) is ICG is a negatively charged, amphiphilic, water-soluble but relatively hydrophobic, tricarbocyanine with a molecular mass of 776 Da. Its applications include assessing cardiac output, hepatic function, hepatic and gastric blood flow, and performing ophthalmic and cerebral angiography. Among its numerous uses, it is also employed in surgery for sentinel lymph node biopsy (9, 10).

When ICG is injected into the bloodstream, binds to plasma proteins and emits fluorescence when exposed to near-infrared (NIR) light. The Firefly® system integrates NIR light sources and specialized cameras into the da Vinci system's vision setup. In recent years, intraoperative ICG fluorescence imaging in combination with Da Vinci robot has shown unique advantages in urological surgical procedures (10).

Lipiodol, an iodized oil-based contrast agent, was the first opaque contrast medium developed for radiological imaging. Since its invention in 1901, it has revolutionized diagnostic and therapeutic fields, adapting to evolving clinical needs. Today, Lipiodol is primarily used for other indications, such as the treatment of hepatocellular carcinoma (HCC) via conventional transarterial chemoembolization (cTACE), acute arterial hemorrhage, cerebral or peripheral arteriovenous malformations, and mapping tumor lesion extension (11).

A mixture of these two agents can be administered to patients via injection into the prostatic arterial vessels. This mixture is subsequently taken up by loco-regional lymph nodes that drain neoplastic prostatic tissue. The fluorescence emitted by these lymph nodes could serve as a potential marker of loco-regional metastatic involvement.

This study aims to evaluate the potential of this technique by comparing intraoperative fluorescence with the results of pathological examination. If validated, this technique could obviate the need for iliac-obturator lymphadenectomy in patients who do not require it, thereby avoiding associated complications.

## 2 Materials and methods

### 2.1 Patient selection

To assess the feasibility and effectiveness of the proposed technique, the study included only patients diagnosed with high-risk or unfavorable intermediate-risk PCa scheduled for radical prostatectomy (RP) with ePLND. Inclusion criteria were independent factors such as age, body mass index (BMI), International Prostate Symptom Score (IPSS), International Index of Erectile Function (IIEF-5), International Society of Urological Pathology (ISUP) grade, clinical T and N stage (excluding cT4), prostate volume, or seminal vesicle involvement on imaging. For all patients with unfavorable intermediate-risk PCa, the Briganti score 2019 was calculated to confirm the indication for ePLND. Preoperative staging included mpMRI, bone scintigraphy, whole-body CT, and PSMA-PET to assess loco-regional disease extent, lymph node involvement, and the presence of distant metastases.

### 2.2 Angiography and prostatic artery embolization

All enrolled patients underwent angiography and selective embolization of the prostatic arteries 24–48 h prior to the scheduled surgery. A Foley catheter with a balloon inflated with 10 ml of a solution consisting of Iodinated contrast agents and sterile saline (1:3 ratio) was placed to serve as an anatomical marker during the angiographic procedure. This aided in the visualization of the prostatic lodge, vesical arteries, and prostatic arteries, while accounting for potential anatomical variations.

The angiographic procedure was performed via femoral approach. After the right arterial femoral puncture a 4-Fr vascular sheath was introduced and flushed with heparinized saline. A 0.035-inch guidewire was used to guide a 4-Fr catheter (RIM or Renal Double Curve) under fluoroscopic guidance. Once the diagnostic catheter was positioned in the internal iliac artery, selective digital subtraction arteriography (DSA) was performed to better assess the prostate's blood supply. The 4-Fr diagnostic catheter was positioned at the common internal iliac trunk to ensure that no branches of the prostatic artery arising from both the anterior and posterior divisions were missed. The optimal view to identify the prostatic artery and its possible accessory branches was the 20°–50° ipsilateral oblique angle. Alternatively, an arterial rotation computed tomography (CT) angiography scan (cone-beam CT) could be used to assess the vascular patterns (2 ml/s, with a 4–6 s delay). Once the prostatic artery was catheterized, 100–200 mg of nitroglycerin diluted in saline was injected to prevent vasospasm and enhance the artery's diameter to facilitate distal catheterization.

**Figure 1:**
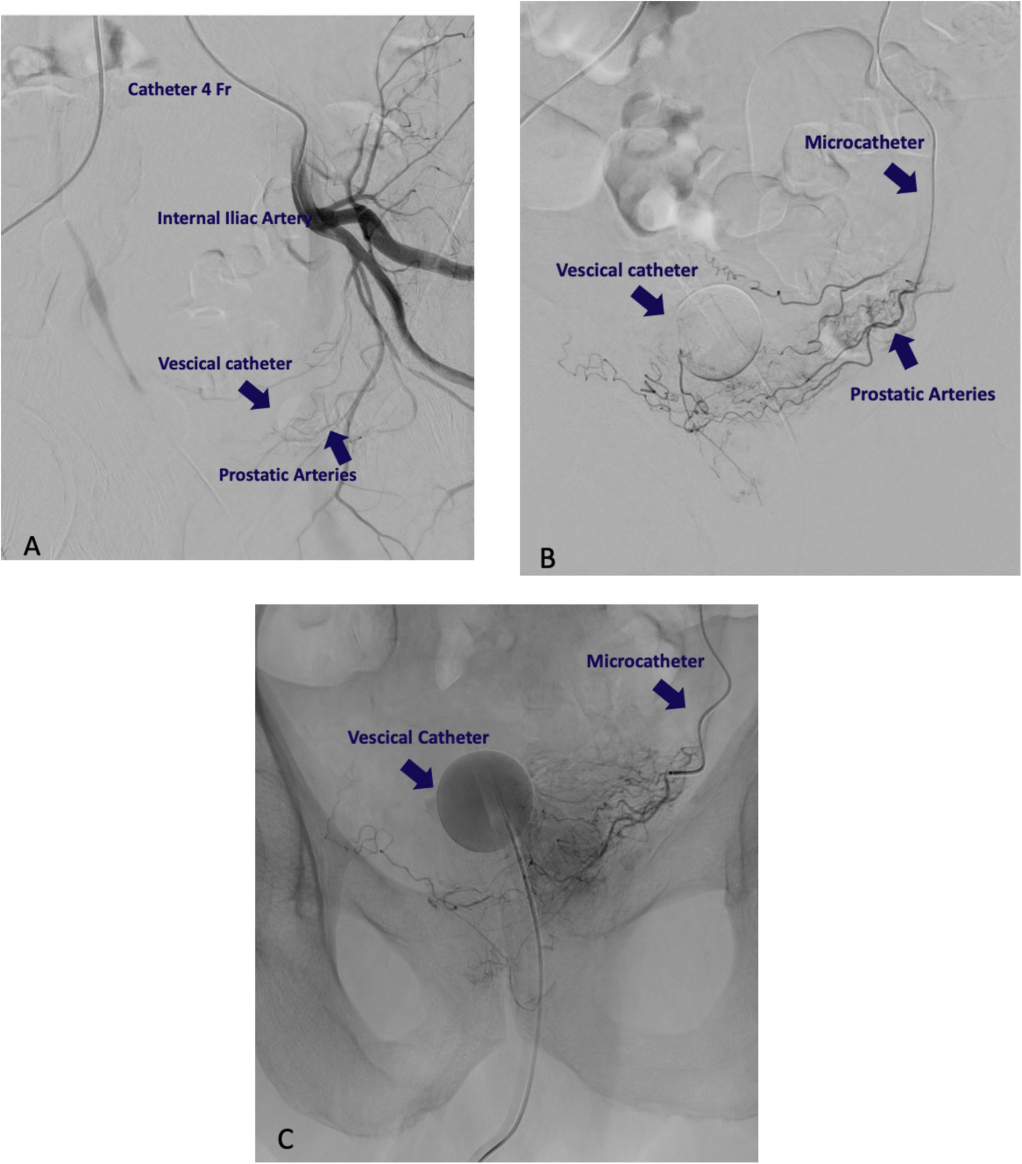
Radiological intervention procedure. **(A)** Catheterization of internal iliac artery. **(B)** Identification of prostatic artery. **(C)** Embolization with ICG-Lipidiol. The slowdown of the injection flow and the contrast opacification of the prostatic homolateral side confirm the proper embolization.

Superselective catheterization of the prostatic arteries was achieved using a coaxial technique with a 2.4 Fr microcatheter (Progreat) and a 0.014-inch guidewire. Correct catheterization was confirmed through arteriography showing contrast uptake in the prostate gland. Once the microcatheter position was confirmed, slow direct injections of a fluoro-embolizing solution were performed. The solution was prepared by mixing in a 1:1 ratio Lipiodol Ultrafluid and indocyanine green (ICG) powder (25 mg/5 ml), diluted previously with 5 ml of distilled water. The stasis of the radiopaque solution within the prostate gland indicates the successful completion of the embolization. On average, 1.5 ml of the fluoroscopic embolizing mixture was used per side. The embolization was then performed on the contralateral side using the same technique. Careful attention was paid to the injection speed and volume of the mixture to avoid spillage into adjacent areas or excessive fluorescence of lymph nodes or the prostate itself, which could compromise procedural outcomes. Unlike similar technical procedures, such as prostatic artery embolization for benign prostatic hyperplasia, the goal here was to release the minimal marking mixture needed to ensure lymphatic drainage into metastatic lymph nodes, providing fluorescence only in these regions. Some steps of the procedure of catheterization and embolization are shown in Figure 1 (Figure 1).

### 2.3 Surgical procedure

**Figure 2:**
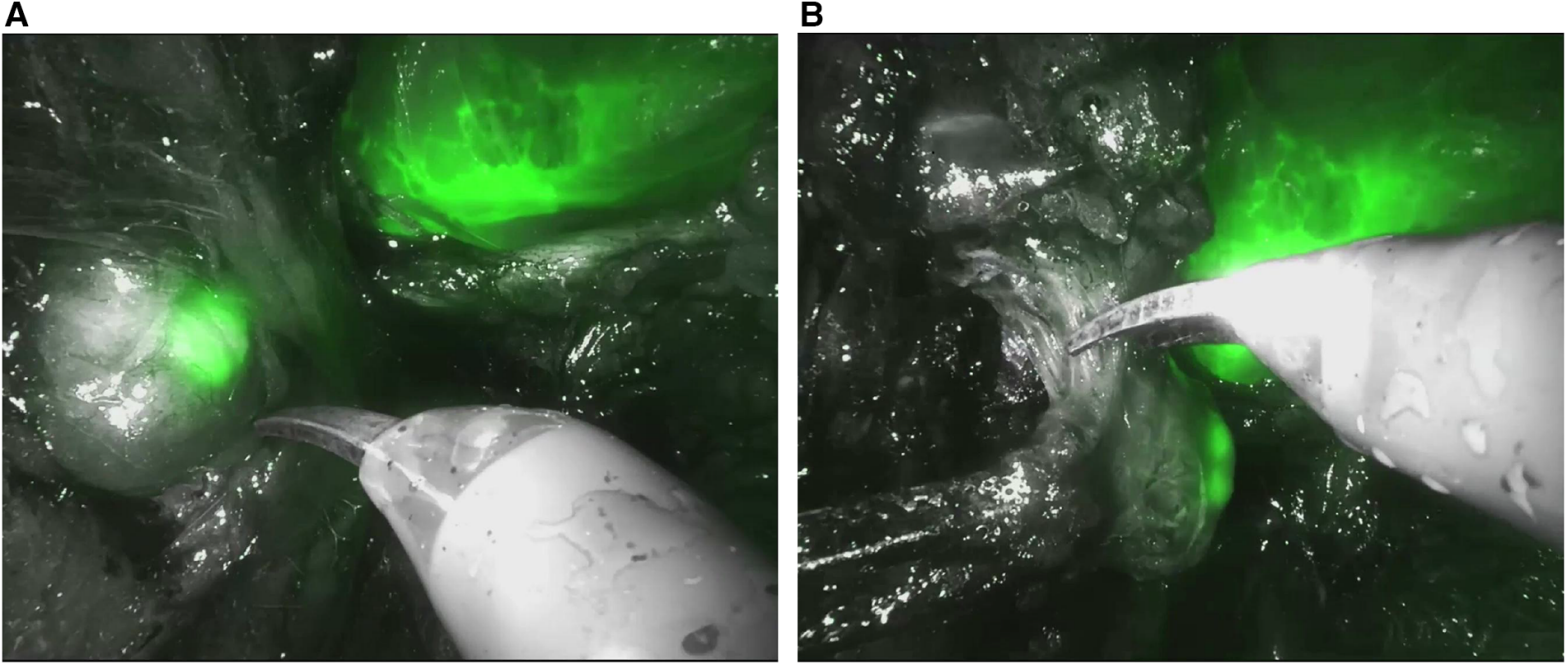
“Firefly” mode integrated into the Da vinci xiTM robotic during robot-assisted radical prostatectomy (RARP). **(A)** Evaluation the fluorescence of loco-regional lymph nodes. **(B)** Excision of loco-regional lymph node.

All patients underwent robot-assisted radical prostatectomy (RARP) with ePLND within 48 h of prostatic artery embolization with ICG-Lipiodol. RARP was performed using either the classical transperitoneal or the Retzius-sparing technique. In both approaches, a bladder neck-sparing technique was applied, with vesicourethral continuity reconstructed using two semi-continuous V-loc 3/0 sutures. Where feasible, considering loco-regional disease conditions and preoperative IIEF-5 values, a nerve-sparing technique was performed to preserve the neurovascular bundles. During ePLND, the “Firefly” mode integrated into the Da Vinci XiTM robotic system was used to evaluate the fluorescence of loco-regional lymph nodes (Figure 2). Lymph node packets from both sides were separately captured in endobags and sent for pathological examination. Fluorescent-positive nodes were separately retrieved for dedicated pathological analysis. Postoperative histopathological examination determined the pathological stages pT and pN.

### 2.4 Sample

Between October 12, 2023, and October 10, 2024, 10 patients underwent RARP with fluorescence-guided ePLND. Surgical indication was based on EAU guidelines following risk stratification of prostate disease and uro-oncological counseling. All study candidates read, understood, and signed informed consent forms for both surgical and angiographic procedures.

### 2.5 Parameters

Recorded data included age, Body Mass index (BMI), age-adjusted Charlson Comorbidity Index (CCI), American Society Anesthesiology score (ASA score), prostate volume, preoperative International Prostatic symptoms score (IPSS), IPSS-Quality of life (QoL), and International Index of Erectile Function (IIEF-5) scores. Based on staging exams, clinical disease stage (TNM system), mpMRI findings, Index Lesion characteristics (including PI-RADS v2 or v2.1 score), PSMA-PET lymph node findings, and preoperative biopsy, the International Society of Urological Pathology grading (ISUP) were documented.

Procedure details such as angiographic procedure duration, volume of embolizing solution used, RARP type, surgical duration, intraoperative Firefly fluorescence findings, final histopathological results, and short-term (within 15 days) postoperative complications graded by the Clavien-Dindo system were also recorded.

### 2.6 Statistical analysis

Continuous variables were expressed as median and interquartile range (IQR; 25th–75th percentiles). Categorical variables were reported as absolute numbers and percentages. Given the study's aim, patients were not classified into sub-cohorts, and comparisons of continuous or categorical variables were not performed.

Bivariate correlation analysis (Pearson and Spearman) was conducted to assess associations between continuous and categorical variables. Divergent negative associations were considered for rho (*R*) < 0, while convergent positive associations were identified for *R* > 0. Correlation strength was categorized as follows: 0.00–0.19 (very low), 0.20–0.39 (low), 0.40–0.59 (moderate), 0.60–0.79 (high), 0.80–1.00 (very high). Results with *p*-values <0.05 were deemed statistically significant.

Statistical analysis was performed using SPSS Statistics, version 25.0 (IBM Inc., Armonk, NY, USA). A phi coefficient was calculated to establish the the degree of correlation between two binary variables, the detection of green fluorescence by Firefly system and the positivity of lymph nodes for metastatic invasion at the anatomo-pathological analysis.

## 3 Results

A total of 10 patients were included in the study. Median age recorded was 67 years (IQR: 58.75–71), with a BMI of 26.07 kg/m^2^ (IQR: 24.31–26.81) and a CCI (adjusted for age) of 3 (IQR: 2–3.25); the majority of patients (80%) were classified as ASA 2. The prostate volume had a median value of 35.50 ml (IQR: 26.25–44.50), with a total PSA of 10.75 ng/ml (IQR: 3.76–14.06) and a PSA density of 0.25 ng/ml (IQR: 0.13–0.52), which is above the current cutoff used in the EAU guidelines to indicate the need for prostate biopsy. The results of the descriptive analysis of the clinical-demographic parameters are presented in Supplementary material Table S1.

The preoperative staging parameters are shown in Supplementary material Table S2; of all patients, 20% presented with a cT3 stage at the time of prostate biopsy, indicating locally advanced disease. 20% of patients had a cN1 stage based on preoperative imaging, consistent with pre-surgical metastatic lymph node involvement. On preoperative mpMRI, the majority of patients (80%) had organ-confined disease. No patients showed seminal vesicle invasion on preoperative imaging. The median size of the Index Lesion was 17 mm, with a predominance in the peripheral zone of the prostate (80%). In 20% of cases, multiple suspicious lesions were identified on mpMRI. Following prostate biopsy, prostate tumors were classified as ISUP 3, 4, and 5 in 40%, 40%, and 20% of cases, respectively.

The results of the descriptive analysis of the intraoperative and postoperative parameters are shown in Supplementary material Table S3. The procedure for angiography and embolization of the prostate arteries with the ICG-Lipiodol solution had a median duration of 85.50 min. The median amount of ICG-Lipiodol used for the angiographic procedure was approximately 3 ml.

In most cases (70%), RARP was performed with a Retzius-sparing technique (12), considering the relatively small prostate volumes. The Firefly mode showed positive fluorescence for loco-regional lymph node packets in 30% of cases; specifically, it should be noted that positive fluorescence did not involve an entire lymph node packet, but rather individual lymph nodes that were excised and sent separately using the sentinel lymph node technique.

The analysis of the lymph node packets revealed metastatic involvement in 20% of patients. Again, it should be noted that lymph node positivity was restricted to the lymph nodes with positive fluorescence in Firefly mode. Within the same lymph node packet, during ePLND, some lymph nodes exhibited positive fluorescence, were analyzed separately, and confirmed as metastatic upon dedicated histopathological examination, while non-fluorescent lymph nodes were found to be negative for metastatic involvement.

**Figure 3:**
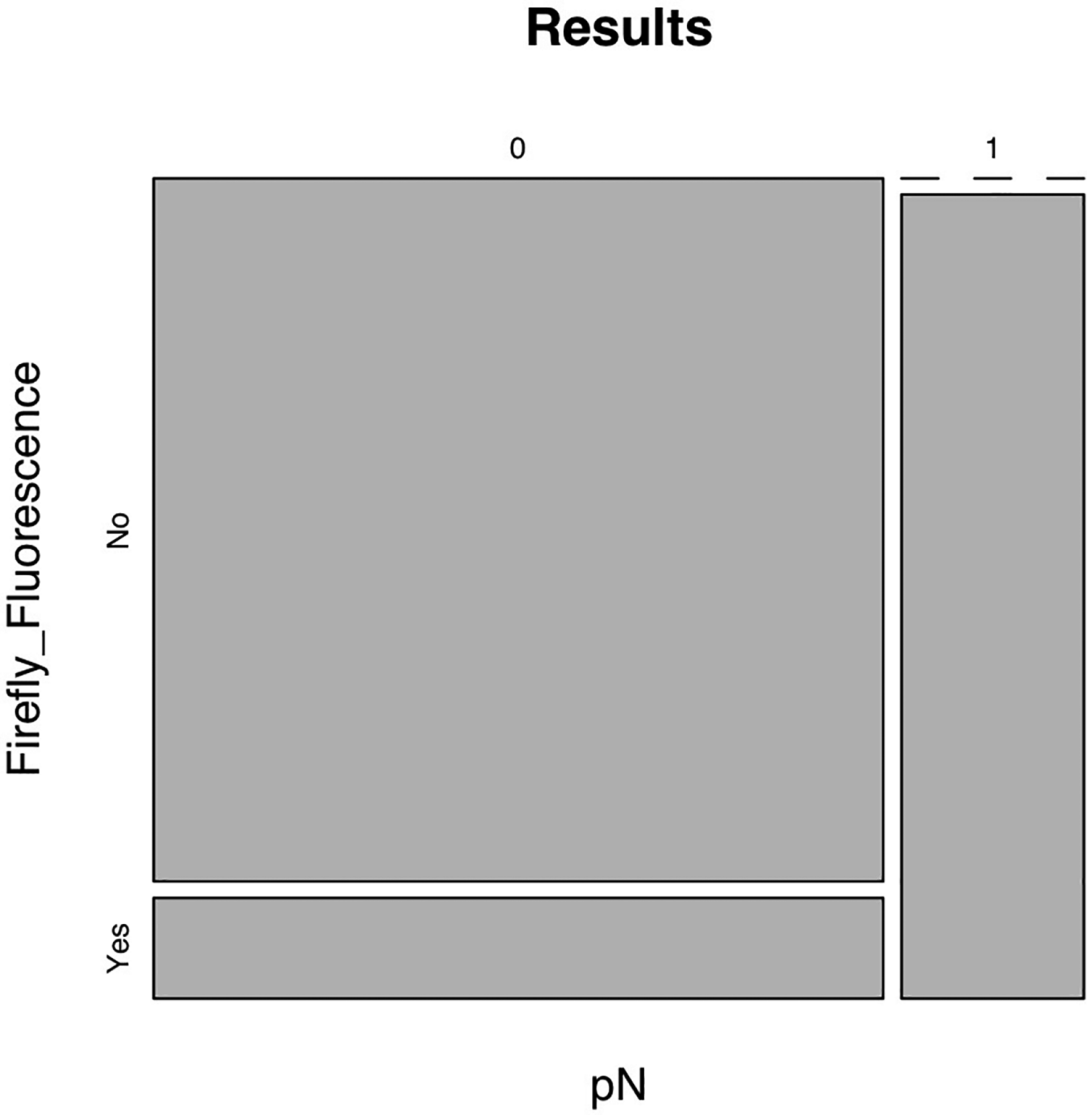
Representation of the association between the pN and firefly fluorescence.

The concordance assessed by phi correlation coefficient was 0.70, with a sensitivity of 100% (95% confidence interval) (Figure 3), confirmed by the absence of false negative results obtained when using Firefly system.

## 4 Discussion

Currently, there is no diagnostic technique that can provide absolute certainty in confirming the potential metastatic involvement of pelvic lymph nodes in patients with PCa. Despite advancements in imaging modalities such as mpMRI, computed tomography (CT), and positron emission tomography (PET), challenges remain in accurately staging PCa, particularly due to the limitations of size-based criteria for individual lymph nodes and the detection of micro-metastases. These limitations persist as critical factors influencing the accuracy of current imaging techniques in oncological staging.

For this reason, the current 2024 EAU guidelines recommend performing ePLND in patients with high-risk, very high-risk, and unfavorable intermediate-risk prostate cancer (based on dedicated scoring systems) to assess the likelihood of metastatic lymph node involvement.

However, the execution of ePLND is not without complications, which may include vascular, nerve injuries, and postoperative lymphocele formation. Over the years, efforts have been made to modify the indications for ePLND to evaluate the actual necessity of the procedure in PCa patients. Although a true sentinel lymph node technique is not yet available in the treatment of PCa, alternatives have been explored to achieve similar results.

One of the first studies to evaluate the potential of the sentinel lymph node technique in PCa dates back to 2016, with the administration of ICG via a transperineal approach to the prostate gland (13). The results of the study showed high lymph node staging in 97% of cases, along with a high negative predictive value that would allow for the avoidance of ePLND, provided there is accurate intraoperative fluorescence analysis of the lymph nodes.

Subsequently, a 2017 meta-analysis gathered evidence from 10 clinical trials on sentinel lymph node techniques with Indocyanine green application in bladder and prostate cancers. Despite the high detection rate of the proposed technique, the specificity in predicting lymph node invasion remained low, further burdened by a high selection bias in the included studies (14).

A more recent study in 2021 evaluated the efficacy of ePLND using a hybrid sentinel lymph node technique, combining a radiotracer and indocyanine green. The use of this technique improved the detection rate of positive lymph nodes while reducing the recurrence risk and optimizing the patient's therapeutic management (15).

Similar results were confirmed in a 2023 study, where the hybrid tracer composed of ICG and a 99mTc improved the positive predictive value for detecting metastatic lymph nodes (16).

In our study, we aimed to investigate an alternative within the currently available sentinel lymph node techniques for PCa. Through embolization of the prostate arteries with the ICG-Lipiodol mixture, selective tracer uptake by the prostate tumor is expected; the addition of Lipiodol would allow the mixture to be retained and transported to the loco-regional lymphatic drainage, resulting in its localization in the iliac-obturator lymph node stations for a variable period of 24–48 h.

Although our study was limited by a small sample size, the results appear promising, indeed, based on the findings of our study, we propose that a strong correlation exists between the detection of green fluorescence by Firefly system and the positivity of lymph nodes by anatomo-pathological analysis, supporting the potential use of the proposed technique to ensure that the lymph nodes excised are indeed those that are histologically confirmed as metastatic.

Notably, during ePLND, not all lymph nodes within the same iliac-obturator packet exhibited uptake of ICG-Lipiodol—only a subset of them. This result, at least speculatively, raises the possibility that metastatic lymph nodes may absorb the tracer more avidly than reactive lymph nodes, allowing for their selective identification. Only one patient showed positive fluorescence with negative histology; upon analyzing this case and consulting with radiology colleagues, it was found that a larger amount of ICG-Lipiodol had been administered to this patient due to the abundant prostatic vascular network.

However, it is possible that the excessive tracer administered led to leakage into the loco-regional tissues, resulting in positive fluorescence due to lymphatic leakage. Given the promising potential of the technique under investigation, further data collection and analysis will be necessary to refine the procedure and minimize the possibility of false positives, with the goal of standardizing the technique based on the preliminary results obtained.

## 5 Conclusions

Although preliminary, the results of this study highlight the potential of fluorescence-guided ePLND following the administration of ICG-Lipiodol for improved identification of metastatic lymph nodes during radical prostatectomy for PCa. The proposed technique, if validated in a larger cohort of patients and appropriately standardized, could complement other existing methods for sentinel lymph node identification in PCa, potentially leading to modifications in the indications for ePLND in the near future. Currently, none of the proposed techniques are recommended by the EAU guidelines, and their implementation remains an area of ongoing research and speculation. Furthermore, the study highlights the growing role of interventional radiology in the future of medicine, characterized by multidisciplinary collaboration and precision treatment.

## Data Availability

The original contributions presented in the study are included in the article/Supplementary Material, further inquiries can be directed to the corresponding author.
