# Supplementary material for: Fluorescence-guided lymphadenectomy in robot-assisted radical prostatectomy: the role of interventional radiology
Source: Front Radiol. 2025 Mar 12;5:1548211. doi: 10.3389/fradi.2025.1548211 (PMC11937084; doi:10.3389/fradi.2025.1548211)
Supplement: Supplementary file 1 [file Datasheet1.pdf]

Supplementary Material

Supplementary Table 1. Study sample's summary statistics.

**Table 1: Descriptive table**		**Totale (n. 10)**
**Age (years)**	Median (IQR)	67 (58.75 – 71)
**BMI (kg/m^2)**	Median (IQR)	26.07 (24.31 – 26.81)
**CCI age-adjusted**	Median (IQR)	3 (2 – 3.25)
**ASA, n. (%)**	2	8 (80)
	3	2 (20)
**Prostata volume (ml)**	Median (IQR)	35.50 (26.25 – 44.50)
**Total PSA (ng/ml)**	Median (IQR)	10.75 (3.76 – 14.06)
**PSA-density (ng/cc)**	Median (IQR)	0.25 (0.13 – 0.52)

Supplementary Table 2. Preoperative staging parameters

**Table 2: Preoperative staging parameters**		Total (10 pts)
**Stage T clinic, n. (%)**	cT1c	1 (10)
	cT2a	5 (50)
	cT2b	2 (20)
	cT2c	0 (0)
	cT3	2 (20)

**Stage N clinic, n. (%)**	cN0 / Nx	8 (80)
	cN1	2 (20)
**Stage mpMRI, n. (%)**	Organ-confined	8 (80)
	Extracapsular	2 (20)
	SV invasion	0 (0)
**Index Lesion Dimension (mm)**	Median (IQR)	17 (15 – 32.50)
**Index Lesion Localization, n. (%)**	PZ	8 (80)
	TZ	0 (0)
	CZ	1 (10)
	AZ	0 (0)
	Entire gland	1 (10)
**Multiple lesions, n. (%)**	No	8 (0)
	Sì	2 (20)
**PET/PSMA lymph nodes, n.(%)**	Negative	8 (0)
	Positive	2 (20)
**ISUP biopsy, n. (%)**	3	4 (40)
	4	4 (40)
	5	2 (20)

Supplementary Table 3. Operative and post-operative parameters

**Table 3: Operative and post operative parameters**		**Totale (n. 10)**
**Angiography and embolization (min),**	Median (IQR)	85.50 (57.75 – 93.50)
**Quantity of ICG-Lipidol (ml), R)**	Mediana (IQR)	3 (3- 4)
**RARP, n. (%)**	Classica	3 (30)
	Retzius-Sparing	7 (70)
**Duration of RARP (min),**	Median (IQR)	197.50 (166.25 – 207.50)
**Time of console (min),**	Median (IQR)	137.50 (113.75 – 147.50)
**Duration of ePLND (min),**	Median (IQR)	40 (30- 45)
**Firefly, n. (%)**	Negative	7 (70)
	Positive	3 (30)
**pT stage,, n. (%)**	pT2	5 (50)
	pT3a	2 (20)
	pT3b	3 (30)
**pN stage, n. (%)**	pN0	8 (80)
	pN1	2 (20)
**ISUP, n. (%)**	2	1 (10)
	3	5 (50)
	4	3 (30)
	5	1 (10)
**Clavien, n. (%)**	1	6 (60)

	2	4 (40)
